# Predicting lncRNA–Protein Interaction With Weighted Graph-Regularized Matrix Factorization

**DOI:** 10.3389/fgene.2021.690096

**Published:** 2021-07-16

**Authors:** Xibo Sun, Leiming Cheng, Jinyang Liu, Cuinan Xie, Jiasheng Yang, Fu Li

**Affiliations:** ^1^Yidu Central Hospital of Weifang, Weifang, China; ^2^Huaibei Kuanggong Zong Yiyuan, Huaibei, China; ^3^Geneis Beijing Co., Ltd., Beijing, China; ^4^Qingdao Geneis Institute of Big Data Mining and Precision Medicine, Qingdao, China; ^5^Academician Workstation, Changsha Medical University, Changsha, China; ^6^Department of Thoracic Surgery, The Second Affiliated Hospital of Hainan Medical University, Haikou, China

**Keywords:** lncRNA–protein interaction, weighted graph-regularized matrix factorization, lncRNA similarity, protein similarity, SFPQ, SNHG3, PRPF31

## Abstract

Long non-coding RNAs (lncRNAs) are widely concerned because of their close associations with many key biological activities. Though precise functions of most lncRNAs are unknown, research works show that lncRNAs usually exert biological function by interacting with the corresponding proteins. The experimental validation of interactions between lncRNAs and proteins is costly and time-consuming. In this study, we developed a weighted graph-regularized matrix factorization (LPI-WGRMF) method to find unobserved lncRNA–protein interactions (LPIs) based on lncRNA similarity matrix, protein similarity matrix, and known LPIs. We compared our proposed LPI-WGRMF method with five classical LPI prediction methods, that is, LPBNI, LPI-IBNRA, LPIHN, RWR, and collaborative filtering (CF). The results demonstrate that the LPI-WGRMF method can produce high-accuracy performance, obtaining an AUC score of 0.9012 and AUPR of 0.7324. The case study showed that SFPQ, SNHG3, and PRPF31 may associate with Q9NUL5, Q9NUL5, and Q9UKV8 with the highest linking probabilities and need to further experimental validation.

## Introduction

Long non-coding RNAs (lncRNAs) are closely associated with many key biological processes, for example, immune response, embryonic stem cell pluripotency, and cell cycle regulation (Chen et al., 2016; Agirre et al., 2019; Gil and Ulitsky, 2020). lncRNAs regulate cellular activities to achieve their biological function through interactions with proteins (Chen and Yan, 2013; Zhang et al., 2018b). Therefore, finding potential lncRNA–protein interactions (LPIs) is important to uncover lncRNA-related biological activities. Wet experiments found a few LPIs; however, experimental methods are costly and time-consuming. Thus, computational methods are developed to identify possible associations between lncRNAs and proteins (Bester et al., 2018; Chen et al., 2018).

LPI prediction methods can be roughly classified into two groups: network-based methods and machine learning-based methods. Network-based LPI identification methods integrated various biological data and network propagation methods (Peng et al., 2019). Li et al. (2015) used random walk with restart on the constructed lncRNA–protein heterogeneous network to find LPI candidates. Zhang et al. (2018a) developed a linear neighborhood propagation method to score for lncRNA–protein pairs. Ge et al. (2016), Zhao et al. (2018a), and Xie et al. (2019) applied bipartite network projection recommended methods to compute the association probabilities between lncRNAs and proteins.

Machine learning-based methods mainly contain matrix factorization-based LPI prediction methods and ensemble learning-based LPI prediction methods. Matrix factorization methods have been widely applied to various association prediction areas (Peng et al., 2020). Liu et al. (2017), Zhang T. et al. (2018), Zhao et al. (2018a), and Shen et al. (2019) used matrix factorization methods to predict possible LPIs. Hu et al. (2018) and Zhang et al. (2018b) utilized ensemble techniques and generated ensemble learning frameworks to discover potential LPIs based on the constructed benchmark datasets. Computational methods effectively revealed the possible associations between lncRNAs and proteins. However, the performance obtained by the above methods is limited and can be further improved.

In this study, we first integrated lncRNA similarity, protein similarity, known LPIs. We then developed a novel LPI prediction method based on weighted graph-regularized matrix factorization (LPI-WGRMF). LPI-WGRMF was compared with five state-of-the-art LPI methods [LPBNI, LPI-IBNRA, LPIHN, RWR, and collaborative filtering (CF)] to measure the performance of the proposed LPI-WGRMF method. LPI-WGRMF obtained the AUC value of 0.9057 and the AUPR value of 0.7324. The results showed that LPI-WGRMF is a useful tool for identifying LPIs. Case study analysis suggests that there are possibly joint links between SFPQ and Q9NUL5, SNHG3 and Q9NUL5, and PRPF31 and Q9UKV8.

## Materials and Methods

In this manuscript, we developed an LPI prediction model, LPI-WGRMF. The method can be summarized to three steps. First, experimentally validated LPIs from the NPInter 2.0 database were collected. Second, lncRNA similarity matrix and protein similarity matrix are computed based on the assumption that lncRNAs tend to associate with similar proteins and vice versa. Finally, lncRNA similarity, protein similarity, and LPI matrix were integrated to the weight graph-regularized matrix factorization model for computing the association scores for each lncRNA–protein pair.

### Materials

#### LPI Data

We obtained experimentally validated LPI dataset, which was provided by Zhang et al. (2018a). The dataset contains 4158 LPIs between 990 lncRNAs and 27 proteins after preprocessing. The LPI matrix between *n* lncRNAs and *m* proteins was denoted as *Y*_*n×m*_.

#### lncRNA Similarity Matrix

The sequence and expression information of lncRNAs can be downloaded from the NONCODE database. We computed lncRNA similarity matrix by integrating the sequence similarity, expression similarity, and interaction similarity to the similarity kernel fusion technique.

##### Sequence statistical similarity

Each lncRNA was described a 20-dimensional vector based on the methods provided by Zhang et al. (2018b). Based on the assumption that each vector can be denoted by their k-nearest neighbors, linear neighborhood similarity between two lncRNAs *l_i_* and *l_j_* can be computed and denoted as *s*_*l*,0_(*i*, *j*).

##### Expression similarity

Suppose that the expression profile of the *i*^*th*^ lncRNA can be represented as *e_i_* and thus the expression similarity between two lncRNAs *l_i_* and *l_j_* can be defined as:

(1) $$s_{l,1}\left(i,j\right)=\{\begin{array}{cc} \frac{1}{2}\left(1+\rho_{i,j}\right) & i\neq j\\ 0 & i=j \end{array}$$

where ρ_*i*,*j*_ is the Pearson’s correlation coefficient between two expression profiles *e_i_* and *e_j_* and is defined as:

(2) $$\rho_{i,j}=\frac{cov\left(e_{i},e_{j}\right)}{\sigma \left(e_{i}\right)\sigma \left(e_{j}\right)}$$

where *cov*() denotes the covariance and σ denotes the standard deviation.

##### Interaction profile similarity

Suppose that the interaction profile of the *i*^*th*^ lncRNA can be represented as the *i*^*th*^ row *Y*_*i.*_ Of the LPI matrix *Y*, the interaction profile similarity between two lncRNAs *l_i_* and *l_j_* can be defined as:

(3) $$s_{l,2}\left(i,j\right)=\text{exp}\left(-\frac{1}{\gamma_{l}}{||Y_{i.}-Y_{j.}||}^{2}\right)$$

where

(4) $$\gamma_{l}=\frac{1}{n}\sum_{i1}^{n}{||Y_{i.}||}^{2}$$

where ||⋅|| denotes the 2-norm of a matrix.

#### Protein Similarity Matrix

##### Sequence alignment similarity

The sequences of proteins were downloaded from the SUPERFAMILY database. The alignment score of the *u*^*th*^ protein against the *v*^*th*^ protein can be computed by Blast and be denoted as *b*_*u,v*_. The sequence similarity between two proteins *p_u_* and *p_v_* can be defined as:

(5) $$s_{p,0}\left(u,v\right)=\{\begin{array}{cc} \frac{b_{u,v}}{b_{u,u}} & u\neq v\\ 0 & u=v \end{array}$$

##### Sequence statistical feature similarity

Each protein can be represented as a 504-dimensional vector based on the method provided by Zhou et al. (2020). Linear neighborhood similarity between two proteins *p_u_* and *p_v_* can be computed and denoted as *s*_*p,1*_.

##### Interaction profile similarity

Suppose that the interaction profile of the *u*^*th*^ protein can be represented as the *u*^*th*^ column *Y*_*.u*_ of the LPI matrix *Y*, the interaction profile similarity between two proteins *p_u_* and *p_v_* can be defined as:

(6) $$s_{p,2}\left(u,v\right)=\text{exp}\left(-\frac{1}{\gamma_{l}}{||Y_{.u}-Y_{.v}||}^{2}\right)$$

where

(7) $$\gamma_{l}=\frac{1}{n}\sum_{u=1}^{m}{||Y_{.u}||}^{2}$$

#### Similarity Kernel Fusion

In the above sections, three lncRNA similarity measurements and three protein similarity measurements were proposed. The similarity kernel fusion method provided by Zhou et al. (2020) was applied to integrate this similarity information to compute a more comprehensive similarity.

First, the three lncRNA similarities were normalized as follows:

(8) $$\theta_{l,q}\left(i,j\right)=\frac{s_{l,q}\left(i,j\right)}{\sum_{t=1}^{n}s_{l,q}\left(t,j\right)},\left(q=0,1,2\right)$$

The normalized similarity matrix was denoted as:

(9) $$\Theta_{l,q}={\left\{\theta_{l,q}\left(i,j\right)\right\}}_{n\times n}$$

Second, for an lncRNA *l_i_* and *s*_*l,q*_, the *k* most similar lncRNAs were collected as a set *N*_*l*,*q*_(*i*, *k*) and *s*_*l,q*_ can be normalized in constraint based on the neighborhood information:

(10) $$\varphi_{l,q}\left(i,j\right)=\frac{s_{l,q}\left(i,j\right)I_{l,q,k}\left(i,j\right)}{\sum_{t=1}^{n}s_{l,q}\left(i,t\right)I_{l,q,k}\left(i,t\right)}$$

where

(11) $$I_{l,q,k}\left(i,j\right)=\{\begin{array}{cc} 1 & l_{j}\in N_{l,q}\left(u,k\right)\\ 0 & l_{j}\notin N_{l,q}\left(u,k\right) \end{array}$$

The neighborhood constrained normalized matrix was denoted as:

(12) $$\phi_{l,q}={\left\{\varphi_{l,q}\left(i,j\right)\right\}}_{n\times n}$$

The above three normalized matrices were integrated based on the following iterative process:

(13) $$\begin{array}{c} \Theta_{l,q}\left(\lambda +1\right)=\frac{1}{2}\alpha \left(\phi_{l,q}\sum_{r\neq q}\Theta_{l,r}\left(\lambda \right)\phi_{l,q}^{T}\right)\\ +\frac{1}{2}\left(1-\alpha \right)\sum_{r\neq q}\Theta_{l,r}\left(0\right) \end{array}$$

where α was a weight parameter with 0 α 1, *T* was the transpose of the matrix, λ represented the iterative parameter, and Θ_*l*,*r*_ (0) Θ_*l*,*r*_.

We computed the integrated similarity matrix after *z* rounds of iteration:

(14) $$\Theta_{l}=\frac{1}{3}\left(\Theta_{l,0}\left(z\right)+\Theta_{l,1}\left(z\right)+\Theta_{l,2}\left(z\right)\right)$$

By considering data noise, we defined the following indicator function based on the *k* most similar lncRNAs for each lncRNA:

(15) $$w_{l,k}=\{\begin{array}{cc} 1 & I_{l,0,k}\left(i,j\right)=I_{l,1,k}\left(i,j\right)=I_{l,2,k}\left(i,j\right)=1\\ 0 & I_{l,0,k}\left(i,j\right)=I_{l,1,k}\left(i,j\right)=I_{l,2,k}\left(i,j\right)=0\\ 0.5 & otherwise \end{array}$$

The final lncRNA similarity matrix can be denoted as follows:

(16) $$S_{l,k}={\left\{\vartheta_{l}\left(i,j\right)w_{l,k}\left(i,j\right)\right\}}_{n\times n}$$

where *ϑ*_*l*_(*i*, *j*) is the (*i*, *j*)^*th*^ element in the matrix Θ_*l*_.

### Nearest Neighbor Information

Based on the graph regularization theory, similar lncRNAs should tend to interact with similar proteins and vice versa in an LPI network, and thus we first observe the nearest neighbor information for lncRNAs and proteins. Given the lncRNA similarity matrix *S^l^*, we represented a *p*-nearest neighbor graph *N* as

(17) $$N_{ij}=\left\{ \begin{array}{cc} 1 & j\in N_{p}\left(i\right)\&i\in N_{p}\left(j\right)\\ 0 & j\notin N_{p}\left(i\right)\&i\notin N_{p}\left(j\right)\\ 0.5 & otherwise \end{array}\right.$$

where *N*_*p*_(*i*) denotes the set of *p* nearest neighbors of lncRNA *l_i_*. *N* is applied to increase the sparsify of the lncRNA similarity matrix *S^l^* as

(18) $$\forall i,j\;{\hat{S}}_{ij}^{l}=N_{ij}S_{ij}^{l}$$

Thus, the sparse similarity matrix of lncRNAs can be computed. Similarity, the sparse similarity matrix of protein can be done.

### Low-Rank Approximation

Based on low-rank approximation idea, the LPI matrix *Y* ∈ *ℝ*^*n* = *m*^ can be decomposed into two low-rank latent feature matrices *A* ∈ *ℝ*^*n* = *k*^ (for lncRNAs) and *B* ∈ *ℝ*^*m* = *k*^ (for proteins) by minimizing the following low-rank approximation objective:

(19) $$\min_{A,B}{||Y-AB^{T}||}_{F}^{2}$$

where ||⋅||_*F*_ denotes the Fronbenius norm and *k* is the rank of matrices *A* and *B*, that is, the number of features in *A* and *B*.

We decomposed *Y* ∈ *ℝ*^*n* = *m*^ into *U* ∈ *ℝ*^*n* = *k*^, *S*_*k*_ ∈ *ℝ*^*k* = *k*^, and V ∈ *ℝ*^*m* = *k*^ so that *US*_*k*_*V^T^* is the closest *k*-rank approximation to *Y* where *U* and *V* are matrices with orthonormal columns, *S_k_* is a diagonal matrix, and *k*_*max*_ = min(*n*, *m*). Thus, the feature matrices *A* and *B* can be represented as $A=US_{k}^{1/2}$ and $B=VS_{k}^{1/2}$.

### Graph-Regularized Matrix Factorization

To boost generalization ability and prevent overfitting, we minimize the following GRMF’s objective function by adding Tikhonov and graph regularization terms to the above low-rank approximation:

(20) $$\begin{array}{c} \min_{A,B}{||Y-AB^{T}||}_{F}^{2}+\lambda_{f}\left({||A||}_{F}^{2}{\left|+\left|B\right|\right|}_{F}^{2}\right)+\lambda_{l}\sum_{i,r=1}^{n}{\hat{S}}_{ij}^{l}{||a_{i}-a_{r}||}^{2}\\ +\lambda_{p}\sum_{j,q=1}^{n}{\hat{S}}_{ij}^{p}{||b_{j}-b_{q}||}^{2} \end{array}$$

where λ_*f*_, λ_*l*_, and λ_*p*_ are positive parameters, *a_i_* and *b_j_* are the *i*^*th*^ and *j*^*th*^ rows of *A* and *B*, respectively, and *n* and *m* are the numbers of lncRNAs and proteins, respectively. The first term is used to make the model approximate the matrix *Y*. The second term (Tikhonov regularization) minimizes the norms of *A* and *B*. The third and final terms are lncRNA graph regularization and protein graph regularization, respectively. The two terms are applied to minimize the distance between feature vectors of two neighboring lncRNAs or proteins. Based on graph regularization, the above model can be redescribed as

(21) $$\begin{array}{c} \min_{A,B}{||Y-AB^{T}||}_{F}^{2}+\lambda_{f}\left({||A||}_{F}^{2}{\left|+\left|B\right|\right|}_{F}^{2}\right)+\lambda_{l}Tr\left(A^{T}{ℒ}_{l}A\right)\\ +\lambda_{p}Tr\left(B^{T}{ℒ}_{p}B\right) \end{array}$$

where *Tr*(⋅) denotes the trace of matrix, ${ℒ}_{l}=D^{l}-{\hat{S}}^{l}$ and ${ℒ}_{p}=D^{p}-{\hat{S}}^{p}$ represent the graph Laplacian terms for ${\hat{S}}^{l}$ and ${\hat{S}}^{p}$, respectively, and *D^l^* and *D^p^* are diagonal matrices where $D_{ii}^{l}=\sum_{r}{\hat{S}}_{ir}^{l}$ and $D_{jj}^{t}=\sum_{q}{\hat{S}}_{jq}^{p}$.

To improve LPI prediction performance, we normalize graph Laplacians ℒ_*l*_ and ℒ_*p*_ by ${\tilde{ℒ}}_{l}={\left(D^{l}\right)}^{-1/2}{\tilde{ℒ}}_{l}{\left(D^{l}\right)}^{-1/2}$ and ${ℒ}_{p}=D^{p}-{\hat{S}}^{p}$. Equation (4) can be rewritten as

(22) $$\begin{array}{c} \min_{A,B}{||Y-AB^{T}||}_{F}^{2}+\lambda_{f}\left({||A||}_{F}^{2}{\left|+\left|B\right|\right|}_{F}^{2}\right)+\lambda_{l}Tr\left(A^{T}{\tilde{ℒ}}_{l}A\right)\\ +\lambda_{p}Tr\left(B^{T}{\tilde{ℒ}}_{p}B\right) \end{array}$$

### Weighted Graph-Regularized Matrix Factorization

To prevent unknown lncRNA–protein pairs from affecting the performance of singular value decomposition produced by *Y*, we add a weight matrix *W* into the objective function as follows:

(23) $$\begin{array}{c} \min_{A,B}{||W⊙\left(Y-AB^{T}\right)||}_{F}^{2}+\lambda_{f}\left({||A||}_{F}^{2}{\left|+\left|B\right|\right|}_{F}^{2}\right)+\lambda_{l}Tr\left(A^{T}{\tilde{ℒ}}_{l}A\right)\\ +\lambda_{p}Tr\left(B^{T}{\tilde{ℒ}}_{p}B\right) \end{array}$$

Based on the alternating least square method provided by Ezzat et al. (2016), we can solve the model (6). Let $\frac{\partial L}{\partial a_{i}}=0$ and $\frac{\partial L}{\partial b_{j}}=0$, run alternatingly the following two update rules until convergence:

∀i=1,2,…n,

(24) $$a_{i}=\left(\sum_{j=1}^{m}W_{ij}Y_{ij}b_{j}-\lambda_{l}{\left({\tilde{L}}_{l}\right)}_{i*}A\right){\left(\sum_{j=1}^{m}W_{ij}b_{j}^{T}b_{j}\lambda_{f}I_{k}\right)}^{-1}$$

∀j=1,2,…m,

(25) $$b_{i}=\left(\sum_{i=1}^{n}W_{ij}Y_{ij}a_{i}-\lambda_{p}{\left({\tilde{L}}_{p}\right)}_{j*}B\right){\left(\sum_{j=1}^{n}W_{ij}a_{i}^{T}a_{i}\lambda_{f}I_{k}\right)}^{-1}$$

where ${\left({\tilde{L}}_{l}\right)}_{i*}$ and ${\left({\tilde{L}}_{p}\right)}_{j*}$ are the *i*^*th*^ and *j*^*th*^ rows vectors of ${\tilde{ℒ}}_{l}$ and ${\tilde{ℒ}}_{p}$, respectively.

We can obtain *A* and *B* based on Eqs 7 and 8. Finally, the interaction probability between the *i*^*th*^ lncRNA and the *j*^*th*^ protein can be computed by

(26) $$Y=AB^{T}$$

## Results

### Experimental Settings

We conducted three different fivefold cross validation on the training dataset to set LPI-WGRMF’s parameters, that is, *k* (the rank of matrices *A* and *B*), *p* (the number of nearest neighbors), λ_*l*_, λ_*d*_, *and* λ_*t*_. We set the parameters as *k* ∈ {50, 100}, *p* ∈ {1, 2, 3, 4, 5, 6, 7}, λ_*f*_ ∈ {2^−2^, 2^−1^, 2^0^, 2^1^}, λ_*l*_ ∈ {0, 10^−4^, 10^−3^, 10^−2^, 10^−1^}, *and* λ_*p*_ ∈ {0, 10^−4^, 10^−3^, 10^−2^, 10^−1^}. And we used grid search and found that the best parameter combination is k=50,p=7,λf=0.5,λl=0.3,andλ=p0.005.

### Evaluation Metrics

Precision, recall, f1 score, accuracy, AUC, and AUPR are widely applied to measure the performance of machine learning methods on association prediction. In this study, we used the six measurements to evaluate the performance of our proposed LPI-WGRMF. AUC is the area under the receiver operating characteristics curve. AUPR is the area under precision–recall curve. The other four criteria are defined as follows:

(27) $$Precision=\frac{TP}{TP+FP}$$

(28) $$Recall=\frac{TP}{TP+FN}$$

(29) $$Accuracy=\frac{TP+TN}{TP+FP+TN+FN}$$

(30) $$\text{f}1score=\frac{2*Precision*Recall}{Precision+Recall}$$

where TP and FP denote the predicted true and false number of positive LPIs, respectively, and TN and FN denote the predicted true and false number of negative LPIs, respectively. The experiments were conducted 20 times. The average precision, recall, accuracy, AUC, and AUPR values for 20 times of experiments were computed as the final performance.

### Performance Comparison of LPI-WGRMF and Other Methods

To measure the performance of our proposed LPI-WGRMF method, we compared LPI-WGRMF and five state-of-the-art methods, that is, LPBNI, LPI-IBNRA, LPIHN, RWR, and CF. LPBNI is a bipartite network inference method; LPIHN is a heterogeneous network inference method based on random walk with restart. The two models obtained better prediction performance in the area of LPI identification and are state-of-the-art LPI prediction methods. The experiments were conducted 20 times under fivefold cross validation. The results are shown in Table 1. The best performance in each column (measurement metric) is denoted in bold in Table 1.

**TABLE 1:** The performance of five LPI prediction methods.

Methods	Precision	Recall	Accuracy	F1-score	AUC	AUPR
LPBNI	0.3794	0.4037	0.9573	0.3876	0.8569	0.3302
LPI-IBNRA	0.5093	0.4165	**0.9641**	0.4521	0.8718	0.4351
LPIHN	0.4122	0.2800	0.9412	0.3324	0.8451	0.2299
RWR	0.3617	0.3521	0.9531	0.3543	0.8134	0.2827
CF	0.3033	0.2949	0.9488	0.2965	0.7686	0.2357
LPI-WGRMF	**0.9314**	**0.6391**	0.8906	**0.6493**	**0.9057**	**0.7324**

*The best performance in each column (measurement metric) is denoted in bold.*

Higher precision, recall, accuracy, and AUC denote better performance. From Table 1, we can find that LPI-WGRMF significantly outperformed other five methods in terms of precision, recall, and AUC. Precision computed by LPI-WGRMF was better 59.27, 45.32, 55.74, 61.17, and 67.44% than LPBNI, LPI-IBNRA, LPIHN, RWR, and CF, respectively. Recall computed by LPI-WGRMF was better 36.83, 34.83, 56.19, 44.91, and 53.86%, respectively. F1-score computed by LPI-WGRMF was better 36.83, 30.37, 56.19, 44.91, and 53.86%, respectively. AUC of LPI-WGRMF was higher 5.39, 3.74, 6.69, 10.19, and 15.14%, respectively. AUPR of LPI-WGRMF was higher 54.92, 40.59, 68.61, 61.40, and 67.82%, respectively.

**FIGURE 1:**
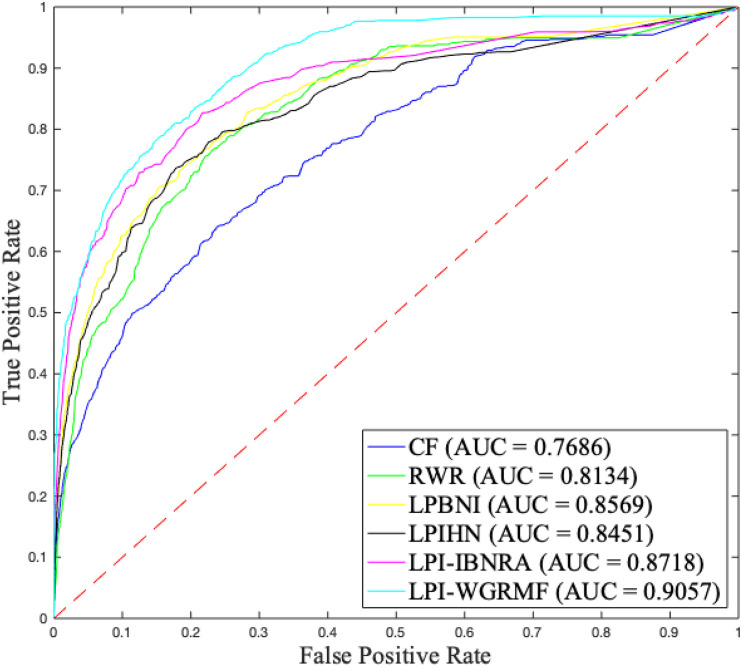
The AUC values of six LPI prediction methods.

**FIGURE 2:**
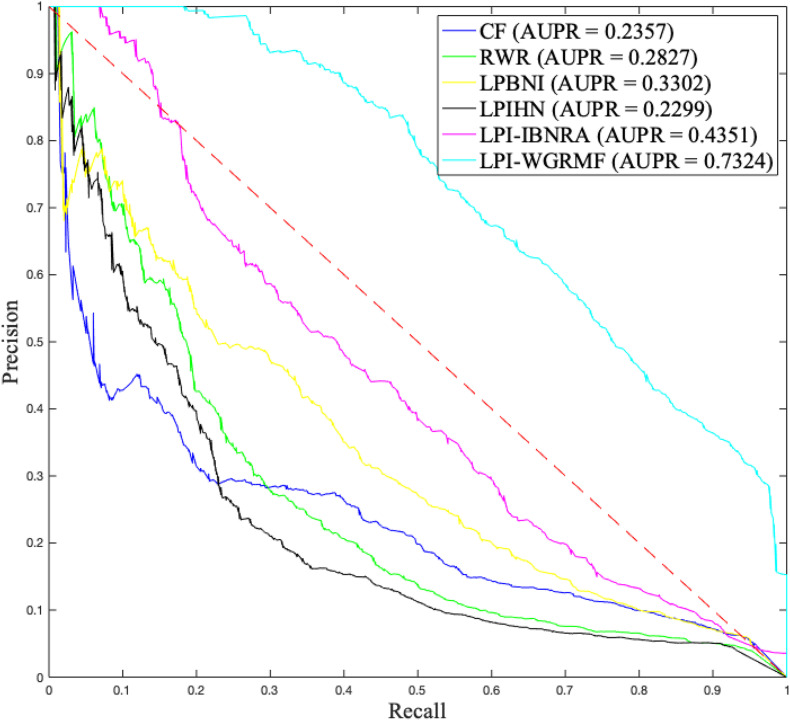
The AUPR values of six LPI prediction methods.

Although accuracy computed by LPI-WGRMF was lower than LPBNI, LPI-WGRMF obtained better precision, recall, and AUC. More importantly, AUC and AUPR are more representative measurement metrics compared with other three evaluation metrics. Thus, AUC and AUPR can be more effectively applied to evaluate the performance of LPI prediction models. LPI-WGRMF is a powerful tool for LPI identification because of its better precision, recall, AUC, and AUPR. Figures 1, 2 demonstrate the AUC and AUPR values obtained by the six LPI prediction methods. The results show that LPI-WGRMF obtained the best AUC value, thereby demonstrating LPI-WGRMF’s powerful LPI prediction capability.

### Case Study

We further conducted four case studies after confirming the performance of LPI-WGRMF. The lncRNAs in the four cases are Splicing Factor Proline and Glutamine Rich (SFPQ), FOrkhead boX protein D2-Adjacent Opposite Strand RNA 1 (FOXD2-AS1), Small Nucleolar RNA Host Gene 3 (SNHG3), and Pre-mRNA-Processing Factor 31 (PRPF31), respectively. We predicted possible LPIs based on lncRNA similarities, protein similarities, known LPIs, and LPI-WGRMF. Table 2 lists the predicted top five proteins associated with the above four lncRNAs.

**TABLE 2:** The top five proteins associated with the four lncRNAs.

lncRNAs	Proteins	Confirmed	LPI-WGRMF	LPBNI	LPIIBNRA	LPIHN	RWR	CF
MTND2P28	Q9NUL5	NO	1	1	4	2	7	2
	O00425	YES	2	2	2	1	1	1
	P26599	YES	3	8	10	11	4	11
	Q07955	YES	4	16	17	18	5	15
	Q9Y6M1	YES	5	3	1	3	2	3
RPI001_1001892	Q9NUL5	YES	1	1	1	1	1	1
	Q07955	YES	2	9	13	15	8	13
	P35637	YES	3	5	5	5	4	5
	P26599	YES	4	15	17	16	9	16
	Q9NZI8	YES	5	4	4	3	5	3
RPI001_1002045	Q9NUL5	YES	1	1	1	1	1	1
	P35637	YES	2	4	2	5	4	5
	Q01844	YES	3	6	6	6	6	6
	P31483	YES	4	9	10	8	7	9
	Q9Y6M1	YES	5	3	4	3	3	3
RP11-169K16.7	Q9UKV8	YES	1	1	1	1	2	1
	Q9H9G7	YES	2	2	4	2	1	7
	Q9UL18	YES	3	7	3	4	4	10
	Q9HCK5	YES	4	6	2	3	3	9
	Q9NUL5	YES	5	5	5	6	5	2

SFPQ is a multifunctional nuclear protein participating in a few cellular activities including RNA transport, apoptosis, and DNA repair. SFPQ is densely associated with several diseases including renal cell carcinoma, Xp11-associated tumor, and dyslexia. More importantly, the expression levels of SFPQ impact on the sensitivity of ovarian cancer cells to PT-induced death (Gao et al., 2019; Pellarin et al., 2020). Table 2 shows that SFPQ has joint connection with Q9NUL5 (ranked as 2). More importantly, the association between SFPQ and Q9NUL5 is ranked as 1 in all other five LPI identification methods. The fact suggests that SFPQ is possibly to link with Q9NUL5.

FOXD2-AS1 is an RNA gene and is abnormally expressed in a variety of malignant tumors. FOXD2-AS1 has close associations with many diseases, for example, nasopharyngeal carcinoma, esophageal cancer, bladder cancer, multiple pterygium syndrome, escobar variant, and ulcerative colitis (Bao et al., 2018; Chen et al., 2018; Su et al., 2018; Huang et al., 2020; Liu et al., 2020). FOXD2-AS1 was predicted to be closely linking with O00425, Q9NZI8, Q9Y6M1, and Q9NUL5, which was ranked as 1, 2, 3, and 4. All these connections were ranked in the top five associations among other five LPI prediction models. Therefore, FOXD2-AS1 is associated with O00425, Q9NZI8, Q9Y6M1, and Q9NUL5.

SNHG3 is a newly found lncRNA and was discovered as a biomarker of malignant cancers, for example, ovarian cancer, hepatocellular carcinoma, colorectal cancer, lung cancer, and glioma (Zhang et al., 2016; Huang et al., 2017; Lu et al., 2019; Liu and Tao, 2020). The results from case study analyses showed that SNHG3 tends to link with Q9NUL5 (ranked as 1) and has highest association scores with the protein in LPNI, BPIHN, and CF. Thus, SNHG3 may be possibly linked with Q9NUL5.

PRPF31 is one retinitis pigmentosa-causing gene. Its genetic variants have joint connections with variation in response to metformin in patients with type 2 diabetes (Kiser et al., 2019). In our predicted results, PRPF31 was found to be densely associated with Q9UKV8 (ranked as 1). More importantly, the association between PRPF31 and Q9UKV8 was identified to be ranked as 1, 1, 2, and 1 in LPBNI, LPIHN, RWR, and CF, respectively. PRPF31 obtained the highest association score with Q9UKV8 in five models.

## Discussion and Conclusion

In this manuscript, we developed a novel method LPI-WGRMF for identifying possible LPIs, based on lncRNA similarity, protein similarity, known LPIs, and weighted graph regularization-based matrix factorization. We first integrated the similarity information and known LPIs as the initial resource. We then proposed a weighted graph-regularized matrix factorization model to compute the association scores for lncRNA–protein pairs.

LPI-WGRMF was compared with five classical LPI methods, that is, LPBNI, LPI-IBNRA, LPIHN, RWR, and CF. Cross-validation experiments were conducted for 20 times. The results showed the powerful performance of LPI-WGRMF. We conducted four case study analyses after confirming the LPI-WGRMF’s accuracy. The results suggest that there are possibly close associations between SFPQ and Q9NUL5, SNHG3 and Q9NUL5, and PRPF31 and Q9UKV8 and need to further experimental validation.

In the future, other sources of LPI-related data may be used to improve the prediction performance, for example, using multiple kernels and designing a multiple kernel learning-based algorithm to effectively integrate the abundant lncRNA and protein information.

## Data Availability Statement

The original contributions presented in the study are included in the article/supplementary material, further inquiries can be directed to the corresponding author/s.

## Author Contributions

FL and JY conceived, designed, and managed the study. XS and LC designed the LPI-WGRMF method, ran LPI-WGRMF, and wrote the original manuscript. JL and CX revised the original draft. XS, JL, and CX discussed the proposed method and gave further research. All authors read and approved the final manuscript.

## Conflict of Interest

JL and CX were employed by the company Geneis Beijing Co., Ltd. The remaining authors declare that the research was conducted in the absence of any commercial or financial relationships that could be construed as a potential conflict of interest.
